# The impact of proton LET/RBE modeling and robustness analysis on base-of-skull and pediatric craniopharyngioma proton plans relative to VMAT

**DOI:** 10.1080/0284186X.2019.1653496

**Published:** 2019-08-20

**Authors:** A. Gutierrez, V. Rompokos, K. Li, C. Gillies, D. D’Souza, F. Solda, N. Fersht, Y.-C. Chang, G. Royle, R. A. Amos, T. Underwood

**Affiliations:** a Department of Medical Physics and Biomedical Engineering, University College London, London, United Kingdom;; b Department of Radiotherapy Physics, University College London Hospitals NHS Foundation Trust, London, United Kingdom;; c Department of Clinical Oncology, University College London Hospitals NHS Foundation Trust, London, United Kingdom

## Abstract

**Purpose:** Pediatric craniopharyngioma, adult base-of-skull sarcoma and chordoma cases are all regarded as priority candidates for proton therapy. In this study, a dosimetric comparison between volumetric modulated arc therapy (VMAT) and intensity modulated proton therapy (IMPT) was first performed. We then investigated the impact of physical and biological uncertainties. We assessed whether IMPT plans remained dosimetrically superior when such uncertainty estimates were considered, especially with regards to sparing organs at risk (OARs).

**Methodology:** We studied 10 cases: four chondrosarcoma, two chordoma and four pediatric craniopharyngioma. VMAT and IMPT plans were created according to modality-specific protocols. For IMPT, we considered (i) variable RBE modeling using the McNamara model for different values of (*α*/*β*)*_x_*, and (ii) robustness analysis with ±3 mm set-up and 3.5% range uncertainties.

**Results:** When comparing the VMAT and IMPT plans, the dosimetric advantages of IMPT were clear: IMPT led to reduced integral dose and, typically, improved CTV coverage given our OAR constraints. When physical robustness analysis was performed for IMPT, some uncertainty scenarios worsened the CTV coverage but not usually beyond that achieved by VMAT. Certain scenarios caused OAR constraints to be exceeded, particularly for the brainstem and optical chiasm. However, variable RBE modeling predicted even more substantial hotspots, especially for low values of (*α*/*β*)*_x_*. Variable RBE modeling often prompted dose constraints to be exceeded for critical structures.

**Conclusion:** For base-of-skull and pediatric craniopharyngioma cases, both physical and biological robustness analyses should be considered for IMPT: these analyses can substantially affect the sparing of OARs and comparisons against VMAT. All proton RBE modeling is subject to high levels of uncertainty, but the clinical community should remain cognizant possible RBE effects. Careful clinical and imaging follow-up, plus further research on end-of-range RBE mitigation strategies such as LET optimization, should be prioritized for these cohorts of proton patients.

## Introduction

In this study, we consider adult base-of-skull sarcoma and chordoma cases plus pediatric craniopharyngioma cases, all widely regarded as priority candidates for proton therapy. Base-of-skull tumors (skull base sarcomas, chordomas and chondrosarcomas) are radio resistant and are particularly challenging to treat due to their proximity and sometimes overlap with organs at risk (OARs) such as the brainstem and optical apparatus. Further, craniopharyngiomas are typically found in pediatric patients who are sensitive to long-term radiation effects in normal tissue, for example effects related to cognitive and endocrinological function [1–3].

It is unsurprising that treatment planning studies demonstrate that for intracranial tumors proton therapy reduces the dose of radiation delivered to the whole brain [4]. Further, across a wide range of base-of-skull tumors, dosimetric studies have reported that proton plans better spare organs at risk such as the brainstem, optical apparatus and cochleae [5–8]. The role of proton therapy in the management of base-of-skull tumors has also been discussed in a number of clinical reviews [4,9,10] which consistently suggest that, relative to photons, protons should be preferred. However, proton therapy remains a limited resource and both proton and photon treatments are continuously evolving via advances such as intensity modulated proton therapy (IMPT) and volumetric modulated arc therapy (VMAT), respectively. Additionally, physical and biological uncertainties are much more complicated for proton plans than for photon plans.

The ‘robustness’ of proton plans to physical and biological uncertainties is a topic receiving substantial attention, as summarized in recent reviews [11,12]. Within commercial proton treatment planning systems (TPSs), physical uncertainties (both geometrical isocenter shifts and uncertainties in Hounsfield Unit (HU) to proton stopping power ratio (SPR) conversion) can not only be analyzed (in terms of dosimetric effect) but also used to drive plan treatment optimizations [12]. For skull base tumors, IMPT plans are known to be sensitive (in terms of both target under-dosage and organ at risk over-dosage) to range and set-up errors [13]. It has been demonstrated that ‘robust optimization’ can be used to mitigate the effects of physical uncertainties for base-of-skull chordomas and chondrosarcomas [14].

However, thus far no clinical TPS has facilitated easy analysis of, or treatment optimization based upon, parameters linked to variable proton biology. A fixed dose-scaling factor or relative biological effectiveness (RBE) of 1.1 has long been adopted as a clinical standard to equate the effects of proton and photon doses across all tissues and biological endpoints [15]. *In vitro* it has been demonstrated, however, that proton RBE is not spatially constant, but dependent on proton linear energy transfer (LET) values, which rise sharply as protons reach the end of their range [16–23]. Proton RBE is also known to depend on the intrinsic biological properties of the irradiated cells and the exact biological endpoint considered (e.g., an endpoint of *in vitro* clonogenic cell survival versus an endpoint of an in-vivo tissue reaction) [24]. Over recent years, there have been a number of published warnings on the possible impact of LET upon clinical treatment plans, including for prostate [25–32], breast [30], thoracic [27,31], liver [32], head and neck [27,31,33], pituitary [27] and various brain tumors [31,32,34–36]. These studies consistently demonstrate that we cannot assume treatment plans to be robust to uncertainties in proton RBE.

For base-of-skull and pediatric craniopharyngioma treatment plans, we first consider the nominal (error-free) dosimetric benefits that IMPT offers over VMAT for up to date implementations of the two techniques. We then investigate the impact of IMPT physical and biological uncertainties, again bringing in comparisons against VMAT. We discuss the impact of these uncertainties in light of a recent European Particle Therapy Network (EPTN) consensus document on radiation dose constraints for organs at risk (OARs) in the treatment of adult brain tumors [37]. Finally, based on our findings, we suggest relevant areas for future work.

## Material and methods

### Patient selection

We studied 10 patients: four chondrosarcoma, two chordoma and four pediatric craniopharyngioma cases. The dose prescriptions were drawn from our VMAT clinical protocols and are outlined in Table 1 (for a fair comparison, dose escalation using protons was not considered). Dose constraints for OARs were based on standard photon clinical practice at our center and are also shown in Table 1.

**Table 1.** Our dose prescriptions and organ at risk dose constraints for VMAT and proton plans, drawn from clinical VMAT protocols at our center.

		Chondrosarcomas and chordoma	Paedriatic craniopharyngioma
Total dose prescription [Gy(RBE)]		65.13	50.4
Number of fractions		39	28
Organ at risk	Volume [cm^3^]	Constraint [Gy(RBE)]	Constraint [Gy(RBE)]
Brainstem	0.1	58	50.4
Spinal cord	0.1	58	50
Optic nerves	0.1	58	50
Optic chiasm	0.1	58	50
Retina		Max <49	Max <45
Lenses		Max <6.5	Max <6
Inner ear		Mean ≤52	Mean ≤44
Parotid		Mean ≤30	

### Units

For photon therapy, the standard unit of absorbed dose is Gy, whereas for proton therapy it is Gy(RBE) [15]. As we analyzed both photon and proton data, including separate notations for both Gy and Gy(RBE) would have become cumbersome at certain points in our manuscript text. At such points, we have simply written Gy(RBE), where an RBE of 1 should be assumed for photons. For protons, a standard RBE of 1.1 should be assumed, unless we specify that we are using variable RBE modeling (with modeling methods as described below).

### Treatment planning

Both VMAT and IMPT treatment planning were performed using the Varian (Palo Alto, California, USA) Eclipse TPS, version 13.6. CTVs were delineated using magnetic resonance imaging (MRI), according to tumor-specific national guidelines. As our main objective was to assess OAR doses for VMAT and IMPT (with and without different uncertainty analyses), planning protocols were tailored to the modality.

### VMAT treatment planning

For VMAT, the prescriptions shown in Table 1 were assigned to a PTV which encompassed the CTV plus a uniform margin of 3 mm. Constraints were applied to Planning Risk Volumes (PRVs) where the raw OARs were expanded by a uniform margin of 3 mm. The VMAT plans we considered were those delivered clinically at University College London Hospitals NHS Foundation Trust: for certain cases, the physicians opted to slightly compromise the OAR dose constraints in order to improve target coverage. For VMAT, a dual 6 MV arc (RapidArc) technique was considered.

### IMPT treatment planning

Multi-field robust optimization was implemented in Eclipse using Nonlinear Universal Proton Optimizer version 13.7.16, which performs a voxel-wise ‘worst-case’ optimization. The ‘worst-case’ (also known as ‘minimax’) approach determines the IMPT plan which is as good as possible for the worst error scenario [12,38,39]. Within the robust optimization, geometrical uncertainties of ±3 mm and range uncertainties of ±3.5% were considered. The magnitude of proton range uncertainty due to HU to SPR conversion was assumed proportional to the distance traversed through tissue (the maximum range of the beam), as routinely considered in clinical proton margin recipes [40].

Proton plans used a 4-field star arrangement, with fixed gantry and couch rotations as specified in Supplementary Table 1. A relatively high number of proton fields (4) was used to reduce the impact of uncertainties associated with any single field and the field arrangement was selected to (i) limit beam passage through heterogeneous regions of the skull and (ii) position the distal fall-off of the beams away from critical organs at risk as far as possible [41]. The beam angles were chosen so that the inferior–anterior–lateral beams were approximately tangential to the optic structures and the superior–lateral beams were not ranging out within the brainstem as shown in Supplementary Figure 1. For these plans, the dose constraints were rigidly applied to the raw OARs, the robust optimization process being used to limit the impact of geometric and range uncertainties. In the remainder of the manuscript, dose reported in the error-free scenario is referred to as the nominal case. Also, note that the plans were optimized using a constant RBE of 1.1, with variable RBE values being calculated post-optimization for the nominal IMPT plans.

Regarding IMPT robustness analysis, 12 scenarios were considered: ±3.5% CT number to SPR conversion uncertainties, combined with ±3 mm shifts of the isocenter in *x*, *y* and *z* directions, as shown in Supplementary Table 2. Where ‘worst case’ IMPT results are reported, these consider the scenario within Supplementary Table 2 that returns the highest value for the OAR dose metric under consideration/the lowest value for the CTV dose metric under consideration. The ‘worst case’ robustness analysis assumes that the same error is realized systematically throughout the course of the treatment.

### Variable RBE using the McNamara model and LET_d_ calculation

Various RBE models have been developed over recent years, with broadly consistent trends [27,34]. For the calculation of a variable RBE in our proton plans, we used the McNamara model [42], a phenomenological model based on the linear quadratic model and a highly comprehensive input database. In this model, a compilation of all RBE experimental measurements available before 2014 (287 experimental data points) was used for a nonlinear regression fit of the RBE as a function of the proton dose (*D_p_*), dose-averaged LET (LET_d_) and the ratio (*α*/*β*)*_x_*. The McNamara model predicts increased RBE values for low (*α*/*β*)*_x_* as well as for high LET_d_. It predicts neither particularly high nor low RBE values relative to other model options [27].

The McNamara RBE model was applied in Matlab using in-house code which extended the CERR open source software [43]. We primarily utilized (*α*/*β*)*_x_* = 2 Gy, a value typically considered for late-reacting brain tissue [37], but we also included data using (*α*/*β*)*_x_* values of 3 Gy and 4 Gy for comparison. For simplicity, we generally considered matched (*α*/*β*)*_x_* values for both normal and tumor tissues. Reliable clinical data on tumor (*α*/*β*)*_x_* values for chondrosarcoma, chordoma and pediatric craniopharyngioma tumors are scarce: a recent literature search [44] found only one relevant study which estimated an (*α*/*β*)*_x_* of 2.45 Gy for chordoma [45]. A much higher tumor (*α*/*β*)*_x_* of 10 Gy was also applied in our consideration of CTV coverage. The LET_d_ used in the McNamara model was calculated using an analytical model based upon work from Sanchez-Parcerisa et al. [46], implemented for each plan directly within Eclipse (using code released to us by Varian Medical Systems of Palo Alto, California, USA). The dose threshold for the LET_d_ calculation script was 0.5% of the plan’s maximum dose value. At the time of writing, the Varian analytical LET_d_ script did not support range shifters: we considered centrally located tumors where range shifters were not required.

### Analysis considering dose constraints from the EPTN consensus

In the recent EPTN manuscript on radiation dose constraints for OARs in the treatment of adult brain tumors, constraints were reported as equivalent dose in 2 Gy(RBE) fractions and (*α*/*β*)*_x_* = 2 Gy was assumed for the brain, brainstem and optic chiasm [37]. To consider our results in light of these published dose constraints [37], we transferred relevant brain, brainstem and optic chiasm constraints to our adult fractionation scheme (as reported in Table 1) using the concept of equivalent biological effective dose (BED) [47].

We used the equivalent BED formula:

(1) $$D_{1}=\frac{D_{2}\left(1+\frac{d_{2}}{\frac{\alpha }{\beta }}\right)}{\left(1+\frac{d_{1}}{\frac{\alpha }{\beta }}\right)}$$

to calculate *D*_1_: the total BED isoeffective dose constraint for our fractionation scheme, from *d*_1_: our 1.67 Gy(RBE) dose per fraction for adult chondrosarcoma and chordoma patients (Table 1).

*D*_2_: each total dose constraint published by the EPTN [37].
*d*_2_: the 2 Gy(RBE) dose per fraction considered by the EPTN [37].
(*α*/*β*)*_x_*: the 2 Gy(RBE) value for (*α*/*β*)*_x_* considered by the EPTN [37].

The resulting constraints are included in Supplementary Table 3. In the EPTN report, a higher dose constraint is proposed for the brainstem ‘surface’, compared to the brainstem ‘interior’. As we considered the EPTN constraints only in our plan analysis, not optimization, for simplicity we considered whether the stricter ‘interior’ constraint was met for our complete brainstem structures.

## Results

### Adult cases: Nominal results assuming a fixed RBE of 1.1

**Figure 1.**
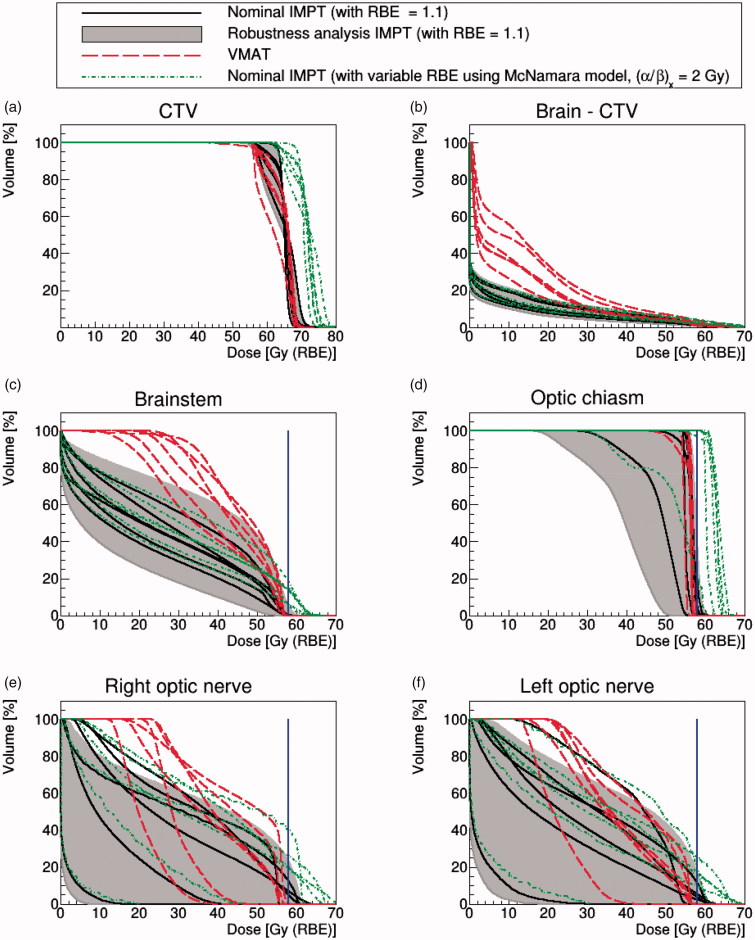
Chordoma and chondrosarcoma cases. DVHs of nominal IMPT (solid black lines) with RBE = 1.1 and VMAT (dash red lines). The gray shaded area represents the robustness analysis IMPT scenarios and the dotted green lines are the McNamara model using (*α*/*β*)*_x_* = 2 Gy. The blue solid line represents the dose constraint at 58 Gy(RBE).

Figure 1 shows the dose volume histograms (DVHs) of the CTV coverage and doses delivered to OARs for all of the adult (chondrosarcoma and chordoma) cases: six patients in total. In this section, we focus on comparing VMAT and nominal, robustly optimized, IMPT with RBE = 1.1.

The mean doses to 95% of the CTV were (88 ± 1)% and (91 ± 4)% of the prescription dose for VMAT and IMPT, respectively (Figure 1(a)). Generally, the IMPT approach delivered improved CTV coverage given the prioritized OAR constraints.

To compare dose levels within the normal brain for the two modalities, we plotted the DVHs of the brain structure minus CTV (Figure 1(b)). As expected, IMPT outperformed VMAT in this regard with a lower mean dose of (5.2 ± 1.3) Gy(RBE), compared to (12.2 ± 2.9) Gy (VMAT).

DVHs for the brainstem are shown in Figure 1(c). We can see that the nominal IMPT scenario achieved a lower mean dose for this OAR: the mean dose for IMPT is (24.5 ± 4.6) Gy(RBE) compared to (42.2 ± 4.0) Gy for VMAT.

However, for the optical chiasm, both approaches resulted in similar nominal dosimetry due to the structure’s small size and the fact that the majority of its volume was located within the target: this OAR was typically irradiated up to our dose constraint without slack for dose sparing (Figure 1(d)). The mean dose to the optical chiasm was (54.9 ± 3.5) Gy(RBE) and (56.0 ± 0.7) Gy for IMPT and VMAT, respectively.

As shown in Figure 1(e,f), the dose to the optic nerves was generally well-controlled relative to our 58 Gy(RBE) dose constraint. However, IMPT better spared the optic nerves: the mean optic nerve dose for IMPT was (24.8 ± 12.8) Gy(RBE), compared to (36.3 ± 7.7) Gy for the VMAT. The higher VMAT mean dose is attributable to the geometrical implementation of arc therapy, where patients' lenses and optic nerves necessarily fell within the radiation path.

A summary of mean and maximum doses for the CTV and OARs are shown in Supplementary Table 4.

### Adult cases: Results considering the IMPT physical robustness analysis and a fixed RBE of 1.1

The physical robustness analysis scenarios are plotted in Figure 1, as a shaded area constrained by the worst and best cases, alongside results for VMAT and nominal IMPT.

Figure 1(a) considers the CTV, where the worst case physical uncertainty scenarios diminished coverage but typically not beyond that achieved by VMAT. In fact, when the worst case was considered, coverage fell from 91 ± 4 to 89 ± 3% of the prescribed dose for 95% of the CTV volume, relative to 88 ± 1% for VMAT.

Concerning the brain-CTV volume (Figure 1(b)), the robust analysis scenarios did not diminish the advantages of IMPT over VMAT.

For OAR sparing, some physical uncertainty scenarios caused our dose constraints to be exceeded for IMPT. For example, when considering the worst-case scenario, our brainstem dose constraint was exceeded for all adult patients (Figure 1(c)). Overall, 60% of the physical robustness scenarios (43 out of 72 different scenarios for the six adult patients) exceeded our brainstem dose constraint. The mean volume of the brainstem irradiated by our dose constraint (for the worst case scenario) was (1.6 ± 0.3) cm^3^, about 10 times higher than the 0.1 cm^3^ maximum volume considered in our clinical protocol. For four out of six patients, our dose constraint for the optic chiasm was exceeded when the worst case scenario was considered (Figure 1(d)). On the contrary, our optic nerve dose constraint to 0.1 cm^3^ was exceeded for just one patient for the worst case of IMPT physical uncertainty (see Figure 1(e,f)).

### Adult cases: Results considering variable RBE modeling using the McNamara model and (α/β)_x_ = 2 Gy

To assess the impact of applying LET_d_ and variable RBE modeling, Figure 1 also shows the DVHs plots for VMAT, nominal IMPT (RBE = 1.1) and IMPT using the McNamara model with (*α*/*β*)*_x_* = 2 Gy. The variable RBE model predicted a substantial increase of dose for both the CTV and the OARs. Such an increase was expected since variable RBE models predict the greatest elevation in RBE (above 1.1) for tissues with low (*α*/*β*)*_x_* ratios [34]. Applying the RBE modeling with (*α*/*β*)*_x_* = 2 Gy, the mean dose to 95% of the CTV volume became 100 ± 3% of the prescribed dose, corresponding to an increase of (5.7 ± 3.0) Gy(RBE) relative to nominal IMPT with RBE = 1.1. However, considering a higher (*α*/*β*)*_x_* value of 10 Gy for the target, the mean dose to 95% of the CTV volume was reduced to only 89 ± 3% of the prescribed dose (less than the nominal IMPT case).

When the variable RBE modeling was applied to the brainstem, our dose constraint was exceeded for all six adult patients, the mean volume irradiated at our dose constraint becoming 4.1 ± 1.8 cm^3^. The variable RBE modeling also resulted in 100% of the optic chiasm volume being irradiated at or above our dose constraint for four of the six adult cases (Figure 1(d)). Similarly, our dose constraint was surpassed for five out of 12 optic nerve volumes (Figure 1(e,f)).

## Pediatric cases: Summary of the results for all modalities and analyses

**Figure 2.**
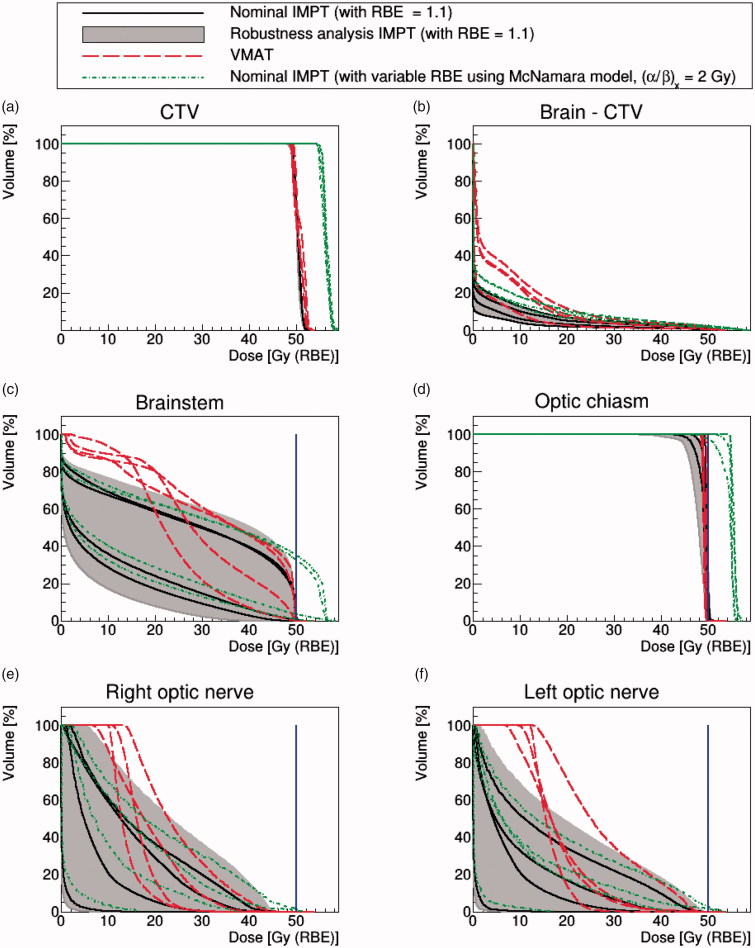
Four pediatric cases. DVHs of nominal IMPT (solid black lines) with RBE = 1.1 and VMAT (dash red lines). The gray-shaded area represents the robustness analysis IMPT scenarios and the dotted green lines are the McNamara model using (*α*/*β*)*_x_* = 2 Gy. The blue solid line represents the dose constraint at 50.4 Gy(RBE) for the brainstem and 50 Gy(RBE) for other OARs.

Results for our four pediatric craniopharyngioma cases are included in Figure 2.

Comparing VMAT against nominal IMPT with constant RBE = 1.1, the target coverage was similar for both, with 95% of the CTV volume receiving 98 ± 1% and 98 ± 0.1% of the prescribed dose for VMAT and nominal IMPT, respectively. CTV coverage remained acceptable for all of the IMPT physical robustness scenarios. Application of the variable RBE model again boosted the dose to the CTV.

As for the adult cases, IMPT spared the normal brain better than VMAT (see Figure 2(b)), regardless of whether robustness analysis or variable RBE modeling was considered. For OARs, however, the variable RBE modeling caused our brainstem and optic chiasm constraints to be exceeded for all patients and our optic nerve constraints to be exceeded for 2/4 patients.

### Detailed results for an example chordoma case

In this section, we focus on one chordoma case which we selected as the CTV coverage achieved was very similar for the VMAT and nominal IMPT treatment plans. However, comparable trends were observed for all other patients.

Supplementary Figure 2 shows the resulting dose-maps for the two approaches, plus the LET_d_ and variable RBE-weighted dose calculations for the IMPT treatment plan. As noted target coverage was similar for both the IMPT and VMAT plans, and as expected the integral doses outside the CTV (within the OARs and normal tissues) were higher for VMAT (see Supplementary Figure 2(a,b)). Application of the McNamara model led to a dose increase of up to 8 Gy(RBE) outside the CTV volume, particularly at the edge of the brainstem. Two high intensity spots are visible at each side of the CTV in supplementary Figure 2(d), indicating that the beam arrangement (in this case the four-field star arrangement with beams from the left and right, as shown in Supplementary Figure 1), has a major impact on modeled LET_d_ and RBE maps.

**Figure 3.**
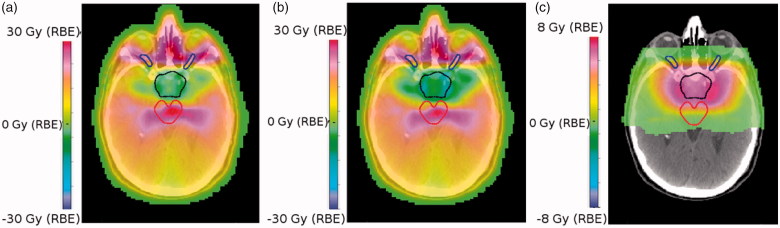
(a) Dose-difference maps for VMAT minus nominal IMPT (RBE = 1.1) (b) VMAT minus nominal IMPT (variable RBE with (*α*/*β*)*_x_* = 2 Gy) and (c) IMPT (variable RBE with (*α*/*β*)*_x_* = 2 Gy) minus IMPT (RBE = 1.1). CTV is outlined in black, the brainstem in red and the optic nerves are in blue.

Figure 3 shows the dose-difference maps for several approaches. For example, Figure 3(a) illustrates VMAT versus nominal IMPT (RBE = 1.1), with differences up to 30 Gy(RBE). These high differences are attributable to the contrasting treatment geometries (360**°** beam angles for VMAT as opposed to four fixed fields for IMPT) and occur mainly in the nasal cavities and around the optical nerves. There is also a substantial dose-difference across the brainstem, reflective of the fact that IMPT reduces the mid/low dose bath to OARs for similar target coverage. Figure 3(b) shows the dose-difference maps for VMAT versus nominal IMPT with variable RBE with (*α*/*β*)*_x_* = 2 Gy. If we compare both IMPT plans (variable RBE vs constant RBE), as shown in Figure 3(c), there is a clear excess in biological dose, particularly at the lateral sides of the CTV.

Supplementary Figure 3 shows the patient’s DVHs for VMAT, nominal robustly optimized IMPT (RBE = 1.1), physical robustness analysis IMPT (RBE = 1.1) and IMPT with variable RBE ((*α*/*β*)*_x_* = 2 Gy). CTV dose coverage was generally preserved regardless of the IMPT physical robustness scenario considered (Supplementary Figure 3(a)). Regarding OARs, our brainstem dose constraint was substantially exceeded for both (worst case) IMPT robust analysis and IMPT with variable RBE (respectively, 1.9 and 2.2 cm^3^ of brainstem received doses in excess of our constraint). 0.2 and 0.6 cm^3^ of the optic chiasm received doses in excess of our constraint for worst case IMPT robustness analysis and IMPT analysis with variable RBE, respectively. The total optic chiasm volume for this patient was in fact 0.6 cm^3^, such that the complete structure received doses in excess of our constraint when variable RBE modeling was considered.

### Summarizing the impact of physical and biological uncertainties on the OARs

**Figure 4.**
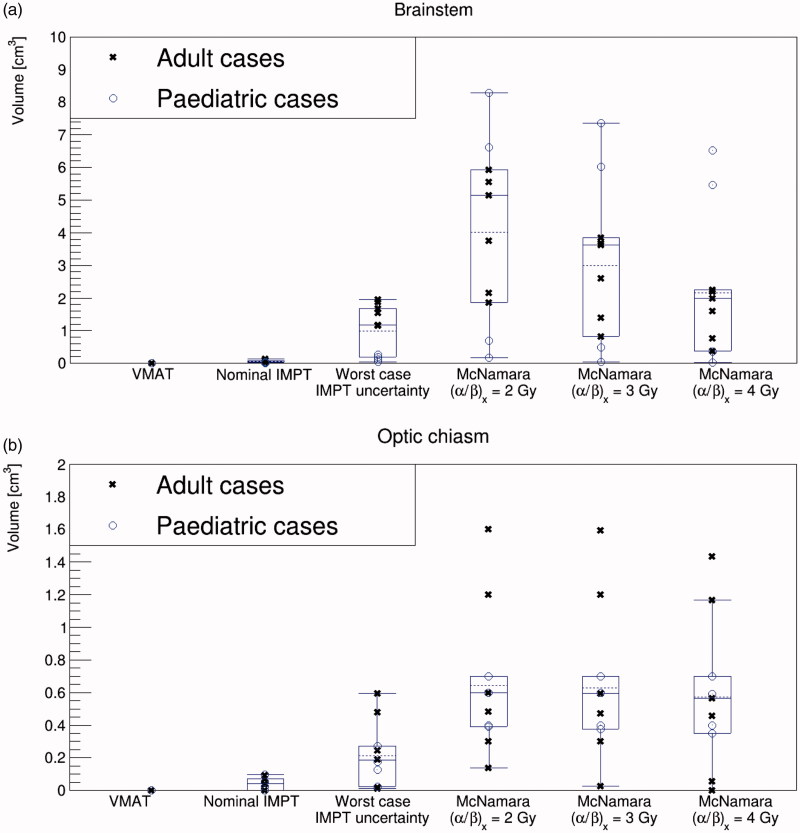
Volume of (a) brainstem and (b) optical chiasm irradiated at or above our dose constraints (58 Gy(RBE) for the adult prescriptions and 50.4 Gy(RBE) for the pediatric prescriptions) for the different treatment modalities and analyses. The mean is drawn as a dashed line, the median as a solid line and the whiskers are drawn to maximum 1.5 × iqr (interquartile range).

Figure 4(a) considers the volume of brainstem irradiated at or above our dose constraints (58 Gy(RBE) for the adult prescriptions and 50.4 Gy(RBE) for the pediatric prescriptions) for the different treatment modalities and analyses. Within the nominal VMAT and robustly optimized IMPT treatment plans, generally less than 0.1 cm^3^ of the brainstem received doses at or exceeding these constraints. However, when the IMPT robustness analysis was performed and the worst-case scenarios were considered (assuming an RBE of 1.1), a spread in the volume arose, with an average of 1.0 ± 0.8 cm^3^ of the brainstem being irradiated to doses at or above our constraint. Further, when biological uncertainties were considered using the McNamara variable RBE model, there was an increase in and an amplified spread in the brainstem volume which received doses exceeding the constraint. The spread was more pronounced for low (*α*/*β*)*_x_*, where a greater increase in biological dose was expected. Average volumes of 4.0 ± 2.7 cm^3^, 3.0 ± 2.4 cm^3^ and 2.2 ± 2.2 cm^3^ received doses in excess of our constraint when (*α*/*β*)*_x_* was modeled as 2 Gy, 3 Gy and 4 Gy, respectively.

Figure 4(b) shows the volume of optic chiasm irradiated at or above our dose constraint. We observed a trend similar to that for the brainstem except that, for many patients, decreasing the (*α*/*β*)*_x_* did not impact upon y-axis results. This is because 100% of the optic chiasm was already irradiated at our dose constraint when variable RBE was considered, even for high (*α*/*β*)*_x_* (e.g., 4 Gy). More outliers were observed for this OAR, likely due to its relatively small absolute volume.

For the adult cases, Supplementary Table 5 considers whether the EPTN brain, brainstem and optic chiasm dose constraints [37] (transferred to our fractionation scheme (Supplementary Table 3)) were exceeded for each patient and each scenario. Supplementary Table 5 reinforces the point is that it is the variable RBE modeling, rather than the worst-case IMPT has the strongest impact upon constraint breach/fulfilment.

## Discussion

In this work, we modeled the impact of physical and biological uncertainties on base-of-skull IMPT treatment plans which were produced in the Varian Eclipse TPS using physically ‘robust optimization’. Optimization based upon biologically relevant parameters such as LET_d_ is not yet available in clinical treatment planning systems. Consistent with published studies [25–31] our findings demonstrated that base-of-skull IMPT plans deemed acceptable under the assumption of a fixed RBE (1.1) did not satisfy OAR constraints when variable RBE modeling was applied. We additionally found the impact of variable RBE modeling to be greater than that of the physical ‘worst case’ analysis. Comparisons between the adult and pediatric groups depended on the OAR, likely due to complex interplays between the dose prescriptions, constraints and target/OAR geometries. For example, for the brainstem, the physical robustness problem was more critical for adults than pediatric patients. For the optic chiasm, problems with physical robustness were consistently evident for both groups. Consistently, variable RBE modeling strongly impacted upon volume of these OARs irradiated at or above our dose constraints.

We calculated voxelized LET_d_ maps using an analytical model based upon the work of *Sanchez-Parcerisa* et al [46], using a script released to us by Varian Medical Systems (Palo Alto, CA, USA). For a single pediatric brain tumor, good agreement has previously been presented between variable RBE (McNamara model) weighted DVHs calculated using LET_d_ (i) from the *Sanchez-Parcerisa* analytical model and (ii) from full Monte Carlo simulations [46]. Further work however could usefully investigate the accuracy of the analytical LET_d_ script provided by Varian under a range of clinical circumstances. The suitability, or otherwise, of averaged LET as a surrogate for biological effect also warrants further investigation [48].

Uncertainties in (i) the McNamara RBE model [42] (and its transferability from *in vitro* experiments to real patients) and (ii) (*α*/*β*)*_x_* values, necessarily limit our interpretation of the absolute variable RBEw dose values we present. The trends however, are clear and ‘best guess’ modeling indicates that OAR constraints (e.g., from the EPTN [37]) primarily based on photon radiotherapy will sometimes be exceeded when proton RBE effects are considered. Our results emphasize the pressing need for further research to consider how IMPT plans can be optimized to be robust to both physical and biological uncertainties. The design of base-of-skull IMPT plans which are biologically robust, physically robust and clearly superior to VMAT in terms of OAR sparing, is non-trivial. Worldwide, considerable effort is being applied to the development of new methods for (i) robust IMPT optimization based on physical and biological parameters and (ii) IMPT robustness analyses [48]. ‘Worst case’ uncertainty scenarios (as employed in the commercial TPS we used) are sometimes criticized for being overly conservative [48]: stochastic or probabilistic approaches form an active area of research and a viable alternative [12]. To date, relatively little research has been performed on robust optimization and robustness reporting in photon therapy [49,50]: additional work in this area could facilitate fairer dosimetric comparisons between treatment modalities. Ultimately, inter- and intra-fraction variations in anatomy could also be considered as part of the robustness problem [51]. Thus far, nonclinical proof of principle demonstrations have shown that LET optimization can, in some cases, be implemented at little cost to IMPT physical dose [52–54]. The rationale to further explore and clinically implement LET-based optimization (to push hotspots away from critical OARs at little cost to target dose distributions) is clearly strong for base-of-skull tumors and pediatric craniopharyngioma. Furthermore, IMPT beam configuration is of paramount importance and additional proton beam angles (moves towards proton arc therapy), may be required if IMPT is to outperform VMAT in a truly robust manner.

The limited clinical evidence for variable proton RBE effects within the brain was recently summarized by Lühr et al. [55] who emphasized the need for further ‘clinical’ RBE data to be obtained from *in vivo* RBE studies and patient outcome analyses. It is evident that careful clinical follow-up should be prioritized in prospective patient studies. Additionally, quantitative imaging might prove useful in investigating possible correlations between normal tissue damage and LET/RBE [56].

## Conclusions

When comparing VMAT and nominal IMPT plans for our base-of-skull cohort, the dosimetric advantages of IMPT are clear: IMPT leads to reduced integral dose (especially to the normal brain) and generally to improved CTV coverage given the OAR constraints. Within our physical robustness analysis, some robustness scenarios returned IMPT plans which exceeded dose constraints (both our local VMAT dose constraints and those recently proposed by the EPTN) for small volumes of critical organs of risk, especially the brainstem. However, variable RBE-weighted dose analyses predicted even more substantial dose-boosts/hotspots within the brainstem and optical chiasm.

In conclusion, both physical and biological robustness analyses should be considered for IMPT treatment plans for base-of-skull and pediatric craniopharyngioma cases. These analyses can substantially affect the sparing of OARs (e.g., relative to the EPTN constraints) and comparisons relative to VMAT. It is desirable to assess different beam arrangements to minimize possible dose excesses due to both physical and biological uncertainties. All proton RBE modeling is subject to high levels of uncertainty, but the clinical community should remain cognizant of possible variable RBE effects. Further, clinical implementation of end-of-range RBE mitigation strategies (such as LET-based optimization) should be prioritized for base-of-skull tumors, alongside careful clinical and imaging follow-up.

## Supplementary Material

Supplemental Material
