# Supplementary material for: The impact of proton LET/RBE modeling and robustness analysis on base-of-skull and pediatric craniopharyngioma proton plans relative to VMAT
Source: Acta Oncol. 2019 Aug 20;58(12):1765–74. doi: 10.1080/0284186X.2019.1653496 (PMC6882303; doi:10.1080/0284186X.2019.1653496)
Supplement: Supplemental Material [file IONC_A_1653496_SM1530.docx]

*Supplementary Table 1. Field arrangement for proton treatment planning of base of skull and paediatric craniopharyngioma cases.*

| Field name | Gantry rotation [deg] | Couch rotation [deg] |
| --- | --- | --- |
| Field 1 | 280 | 345 |
| Field 2 | 270 | 15 |
| Field 3 | 80 | 15 |
| Field 4 | 90 | 345 |

*Supplementary Table 2. IMPT robustness analysis parameters considering isocenter shifts from patient setup error and calibration curve error generated by Eclipse.*

| Patient setup error | | | Proton range uncertainty due to HU to SPR conversion [%] |
| --- | --- | --- | --- |
| x [mm] | y [mm] | z [mm] |  |
| + 3.0 | 0.0 | 0.0 | + 3.5 |
| + 3.0 | 0.0 | 0.0 | - 3.5 |
| - 3.0 | 0.0 | 0.0 | + 3.5 |
| - 3.0 | 0.0 | 0.0 | - 3.5 |
| 0.0 | + 3.0 | 0.0 | + 3.5 |
| 0.0 | + 3.0 | 0.0 | - 3.5 |
| 0.0 | - 3.0 | 0.0 | + 3.5 |
| 0.0 | - 3.0 | 0.0 | - 3.5 |
| 0.0 | 0.0 | + 3.0 | + 3.5 |
| 0.0 | 0.0 | + 3.0 | - 3.5 |
| 0.0 | 0.0 | - 3.0 | + 3.5 |
| 0.0 | 0.0 | - 3.0 | - 3.5 |

Supplementary Table 3. Adult brain, brainstem and optic chiasm constraints from the EPTN transferred to our fractionation schemes.

| Organ at risk | (α/β)_x_ | *EPTN Dose constraint (EQD2*) [11] | Toxicity | BED isoeffective constraint for our chondrosarcoma and chordoma fractionation scheme (1.67 Gy(RBE) fractions) |
| --- | --- | --- | --- | --- |
| Brain | 2 Gy | V_60 Gy_ ≤ 3cc | Symptomatic brain necrosis | V_65.4 Gy_ ≤ 3cc |
| Brainstem | 2 Gy | Interior D_0.03 cc_  ≤ 54 Gy(RBE) | Permanent cranial neuropathy or necrosis | Interior D_0.03 cc_  ≤ 58.9 Gy(RBE) |
| Optic chiasm | 2 Gy | D_0.03 cc_  ≤ 55 Gy(RBE) | Optic neuropathy | D_0.03 cc_  ≤ 59.9 Gy(RBE) |

*Supplementary Table 4: Mean and Maximum dose of studied structures for adult cases for IMPT and VMAT.*

| Structure | Mean dose [Gy(RBE)] | | Maximum dose [Gy(RBE)] | |
| --- | --- | --- | --- | --- |
|  | IMPT | VMAT | IMPT | VMAT |
| CTV | 65.0 ± 0.1 | 64.3 ± 1.2 | 72.7 ± 2.0 | 69.9 ± 0.9 |
| Brain - CTV | 5.2 ± 1.3 | 12.2 ± 2.9 | 69.3 ± 0.8 | 68.7 ± 0.7 |
| Brainstem | 24.5 ± 4.6 | 42.2 ± 4.1 | 60.9 ± 0.6 | 57.5 ± 0.2 |
| Optical chiasm | 54.9 ± 3.5 | 56.0 ± 0.7 | 58.8 ± 2.0 | 57.2 ± 0.7 |
| Left optic nerve | 26.1 ± 13.0 | 36.3 ± 7.3 | 55.8 ± 12.8 | 54.2 ± 5.6 |
| Rigth optic nerve | 23.5 ± 13.6 | 35.7 ± 8.9 | 50.2 ± 18.1 | 52.1 ± 7.1 |

|  | VMAT | Nominal IMPT | Worst case IMPT uncertainty | McNamara  (α/β)_x_ = 4 Gy | McNamara  (α/β)_x_ = 3 Gy | McNamara  (α/β)_x_ = 2 Gy |
| --- | --- | --- | --- | --- | --- | --- |
| Patient 1 | Yes | Yes | No | No | No | No |
| Patient 2 | Yes | Yes | No | No | No | No |
| Patient 3 | Yes | Yes | No | No | No | No |
| Patient 4 | Yes | Yes | No | No | No | No |
| Patient 5 | Yes | Yes | No | No | No | No |
| Patient 6 | Yes | Yes | No | No | No | No |

1. Brainstem dose constraint (58.9 Gy(RBE)) met at 0.03 cc?

|  | VMAT | Nominal IMPT | Worst case IMPT uncertainty | McNamara  (α/β)_x_ = 4 Gy | McNamara  (α/β)_x_ = 3 Gy | McNamara  (α/β)_x_ = 2 Gy |
| --- | --- | --- | --- | --- | --- | --- |
| Patient 1 | Yes | Yes | Yes | No | No | No |
| Patient 2 | Yes | Yes | Yes | Yes | Yes | Yes |
| Patient 3 | Yes | Yes | Yes | No | No | No |
| Patient 4 | Yes | Yes | Yes | No | No | No |
| Patient 5 | Yes | Yes | Yes | No | No | No |
| Patient 6 | Yes | Yes | Yes | Yes | Yes | No |

1. Optic chiasm dose constraint (59.9 Gy(RBE)) met at 0.03 cc?

|  | VMAT | Nominal IMPT | Worst case IMPT uncertainty | McNamara  (α/β)_x_ = 4 Gy | McNamara  (α/β)_x_ = 3 Gy | McNamara  (α/β)_x_ = 2 Gy |
| --- | --- | --- | --- | --- | --- | --- |
| Patient 1 | No | No | No | No | No | No |
| Patient 2 | No | Yes | No | No | No | No |
| Patient 3 | Yes | Yes | Yes | No | No | No |
| Patient 4 | Yes | Yes | No | No | No | No |
| Patient 5 | No | Yes | No | No | No | No |
| Patient 6 | No | No | No | No | No | No |

(c) Brain-CTV dose constraint (65.4 Gy(RBE)) met at 3 cc?

Supplementary Table 5: Considering whether the newly published EPTN brain, brainstem and optic chiasm dose constraints [37] (transferred to our fractionation scheme (Table 2)) were exceeded for each patient and each scenario.


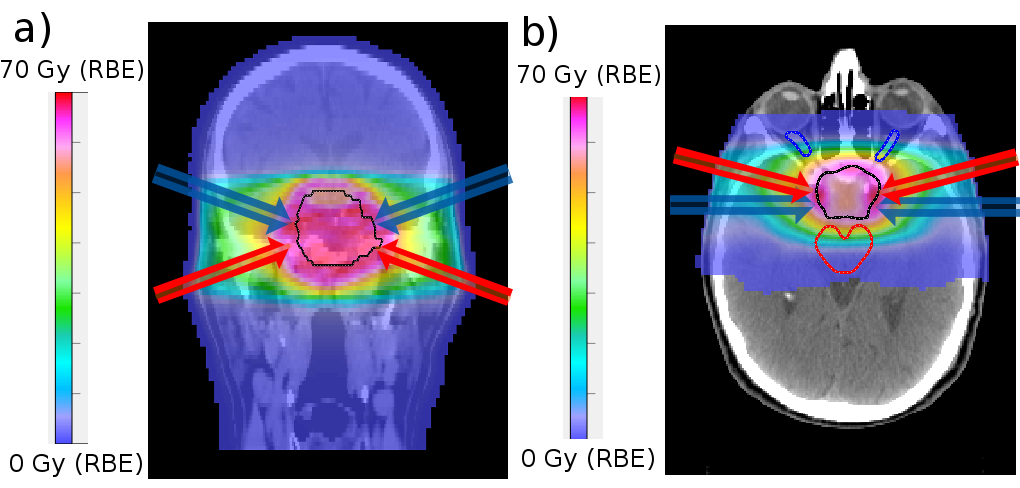


Supplementary Figure 1. Example of beam arrangement showing a view of the a) coronal plane and b) transverse plane of an IMPT treatment plan. The arrows represent the 4-field star beam arrangement: inferior-anterior-lateral beams (red arrows) and superior-lateral beams (blue arrows).


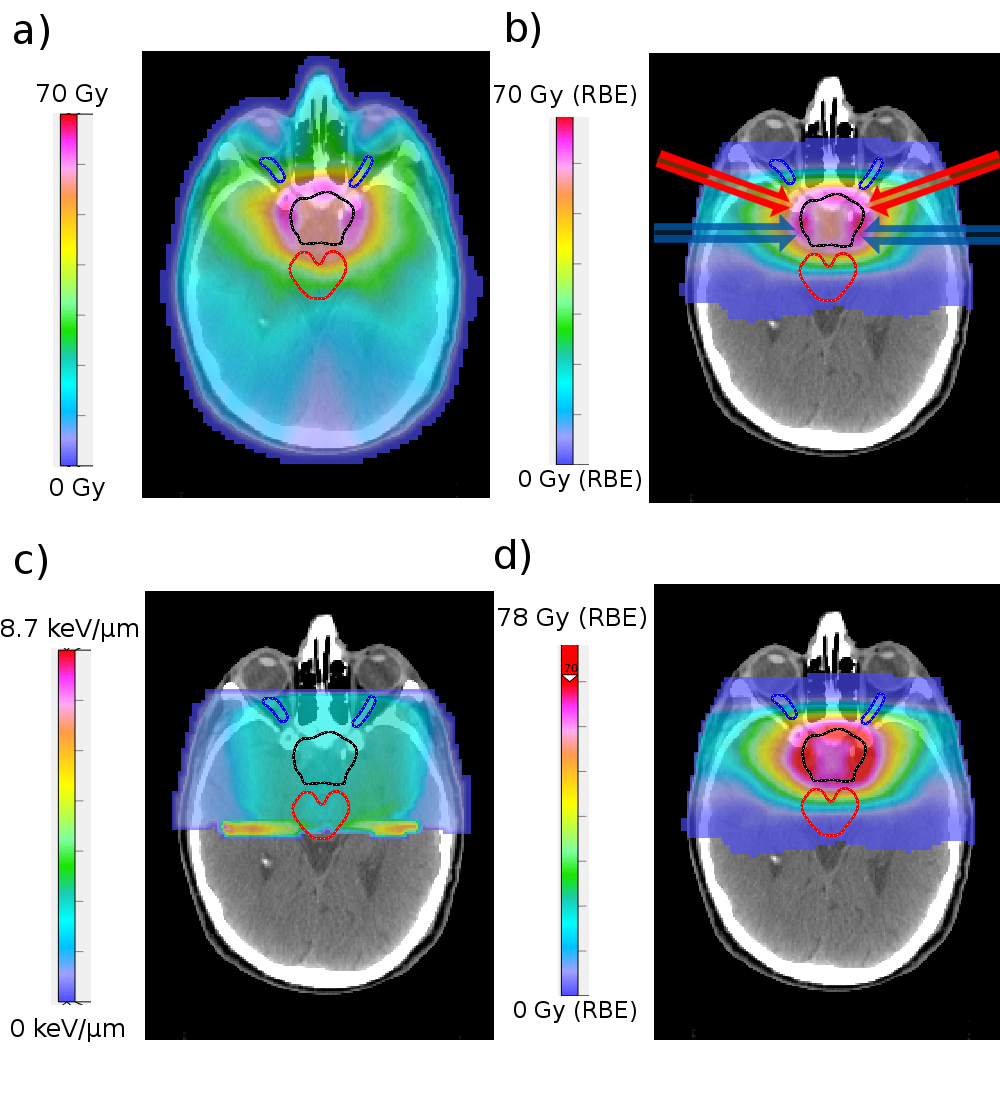


Supplementary Figure 2. a) Dose for VMAT plan, b) Dose for IMPT (RBE =1.1) plan, c) LET_d_ map from IMPT plan and d) IMPT plan using variable RBE McNamara model (α/β)_x_ = 2 Gy. CTV is defined in black, the brainstem in red and the optic nerves are in blue.


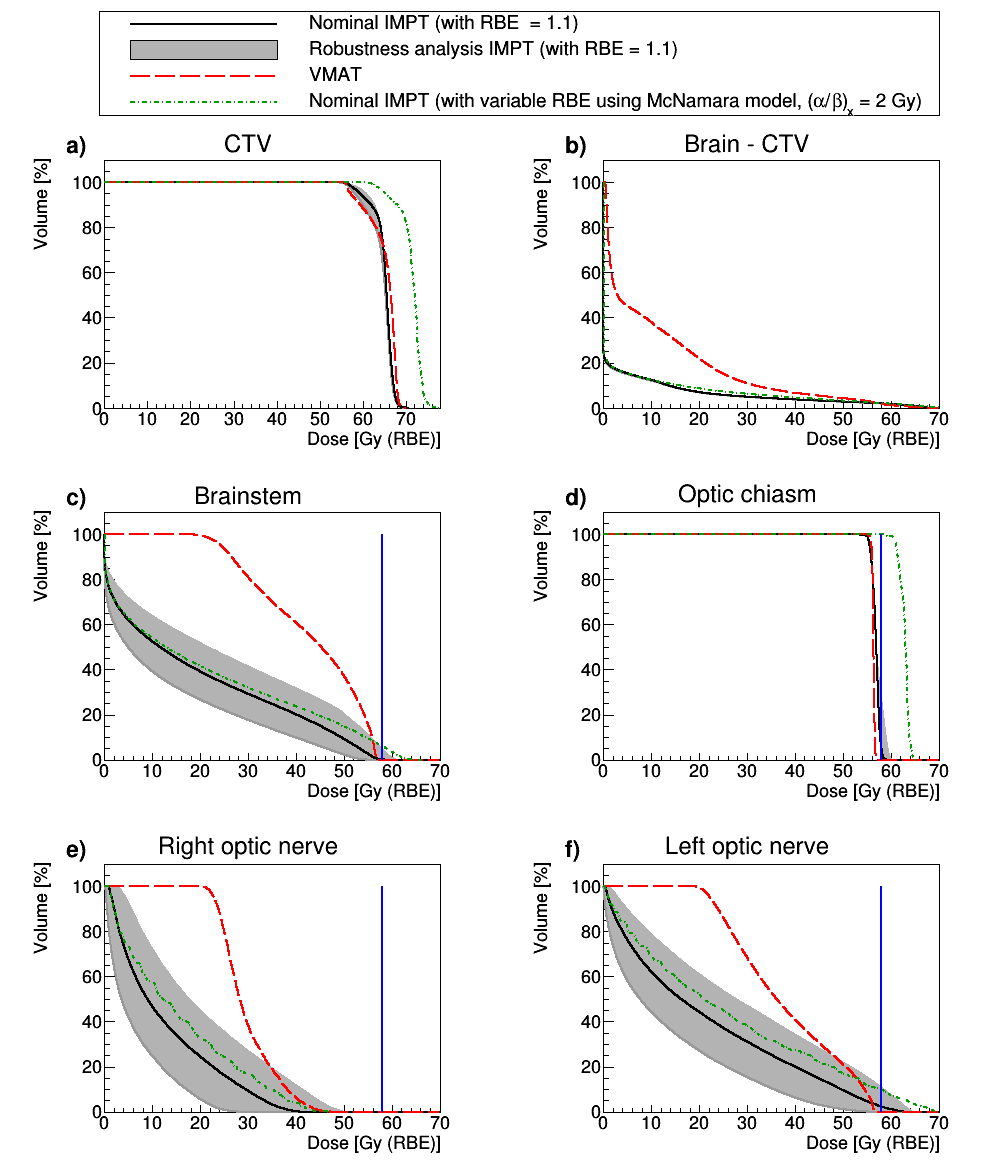


*Supplementary Figure 3. One Chordoma case. DVHs of nominal IMPT (solid black lines), VMAT (dash red lines) and McNamara model using (α/β)_x_ = 2 Gy (dotted green lines). The gray shaded area represents IMPT robustness analysis. The blue solid line represents the dose constraint at 58 Gy(RBE).*
